# Collision Glial Neoplasms Arising in an Ovarian Mature Cystic Teratoma: A Rare Event

**DOI:** 10.1155/2020/7568671

**Published:** 2020-02-03

**Authors:** Abdelrazak Meliti, Bayan Hafiz, Haneen Al-Maghrabi, Abdulrahim Gari

**Affiliations:** ^1^Department of Anatomic Pathology, King Faisal Specialist Hospital and Research Center, Jeddah, Saudi Arabia; ^2^Department of Laboratory Medicine and Pathology, University of Alberta, Edmonton, Alberta, Canada; ^3^Department of Obstetrics and Gynecology, King Faisal Specialist Hospital and Research Center, Jeddah, Saudi Arabia

## Abstract

Germ cell neoplasms represent around 20% of all ovarian tumors. They most frequently affect children and young adults. Mature cystic teratoma is a common benign ovarian neoplasm comprising about 95% and is made up of all three germ cell embryonic layers. By definition, mature cystic teratoma may be derived from any of the three germ cell lines. On the other hand, immature teratomas contain primitive neuroepithelial elements. However, it is quite uncommon in the English literature to have a neuroepithelial glial neoplasm arising in a mature cystic teratoma of an adolescent. Interestingly enough, all published cases described a single type of glial neoplasm arising in mature ovarian teratoma. Herein, the authors discuss a unique case of concomitant occurrence of two different glial neoplasms, namely pilocytic astrocytoma and subependymoma arising in an ovarian mature cystic teratoma. To the best of our knowledge, this is the first reported case with such a distinctive histopathologic finding.

## 1. Introduction

Mature cystic teratoma accounts for approximately 20% of all ovarian neoplasms, and commonly affects the childhood age group [1, 2]. Approximately 88% of cases are unilateral and present clinically with mass compression-related symptoms, hemolytic anemia, flank pain, or virilization [3]. Mature cystic teratoma can coexist with other ovarian neoplasms such as Brenner tumor and thecoma [4]. By definition, mature cystic teratoma should have mature histologic elements from any of the three germ cell components. Grossly, it usually presents as a multilocular mass with cystic components of variable sizes. On microscopic examination, most mature teratomas are lined by mature squamous elements. Blackwell and colleagues found that 100% of these tumors are ectodermal derivatives in origin, 93% are mesodermal, and 71% are endodermal derivatives [5]. Keratin, sebum, hair, and teeth are frequently present.

Additionally, other types of tissues can be seen, such as cartilage, respiratory epithelial tissue, gastrointestinal lining tissue, thyroid, pituitary, neuroendocrine cells, prostate, pancreas, and blood vessels. Prominent lymphoid aggregate with germinal center formation is a differentiating feature of ovarian teratomas associated with autoantibodies against the N-methyl-D-aspartate (NMDA) receptor seen in paraneoplastic syndromes [6]. Among common features seen in mature cystic teratoma are skin appendages and neural tissue. The latter may differentiate into meningothelial tissue and Wagner–Meissner corpuscles structures [7, 8]. “Somatic-type” tumors developing in mature cystic teratoma are uncommon events, accounting for only 2%. Squamous cell carcinoma is the most commonly documented malignant neoplasm [9]. Other less common malignant transformations include melanoma, carcinosarcoma, Paget's disease, glioblastoma, neurocytoma central-type, and neuroblastoma/PNET.

In addition, a wide range of benign tumors has also been reported, such as blue nevus, sweat gland tumors, sebaceous adenoma, glomus tumor, epithelioid hemangioma, and pituitary adenoma [10]. Well-differentiated neuroepithelial components have been rarely documented in the English literature. Nevertheless, astrocytic, ependymal, pilocytic, and oligodendrocytic components can be seen in mature cystic teratoma. The previously reported cases demonstrate only one type of well-differentiated neuroepithelial component. To the best of our knowledge, we present the first case with synchronous well-differentiated glial neoplasms arising in a mature cystic teratoma of a young female.

## 2. Case Presentation

**Figure 1:**
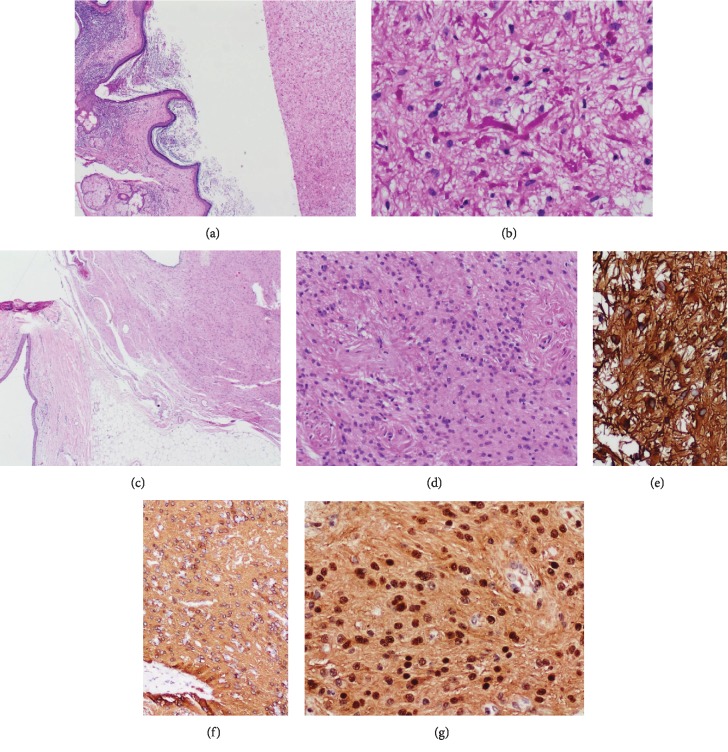
Hematoxylin and eosin (H&E) stains and immunohistochemistry. (a) Low-power field examination shows cystic spaces lined by mature squamous cell epithelium with underling pilosebaceous units and adjacent pilocytic astrocytoma (H&E, 4x). (b) Bipolar cellular neoplasm of low-grade cytology along with tapered corkscrew-shaped brightly eosinophilic hyaline material (Rosenthal fibers) (H&E, 40x). (c) A low-power field demonstrates a separate focus of subependymoma comprised of bland monomorphic cellular proliferation in a dense, fine, glial fibrillary background (H&E, 2x). (d) Clusters of isomorphic nuclei with occasional pseudorosettes (H&E, 40x). (e, f) GFAP revealed strong diffuse staining in the pilocytic astrocytoma and in the subependymoma (f), respectively (40x). (g): S100 revealed strong diffuse staining in the subependymoma (40x).

36-year-old female patient, multiparous (5 + 2) all given birth by spontaneous vaginal deliveries, presented with a history of lower abdominal pain almost a year. The pain was occurring almost every month, on-and-off, with multiple emergency department visits. It was not associated with fever, vomiting, nausea, bowel changes, or urinary symptoms. The pain was not related to her menstrual cycle, however was controlled with analgesic pills. On physical examination, the patient was vitally stable (oral temperature: 36.7°C, pulse rate: 70 bpm, respiratory rate: 20 breaths/min, blood pressure: 113/60, and normal oxygen saturation). Abdominal examination demonstrated a soft and lax abdomen with no masses nor tenderness. Other systemic examinations were unremarkable. Her initial investigation showed a complete blood count (hemoglobin: 10.9 g/dL, WBC: 4.79 × 10^9^/L, and platelet: 219 × 10^9^/L). Her blood electrolytes and hepatic function tests were all within normal ranges. Pelvic ultrasound examination demonstrated an echogenic lesion in the right adnexal region measured 4.1 × 4.2 × 4.9 cm, and caused a significant posterior shadowing. Radiologically, it was most likely of a dermoid cyst. Based on the clinical picture and radiological findings, the patient underwent a right oophorectomy. The surgery went uneventful, and the specimen was sent for histopathology evaluation. Gross examination revealed a cystic mass with an intact smooth outer surface (7.0 × 6.5 × 3.0 cm) attached to the fallopian tube (7.0 cm in maximum dimension). The cyst was multilocular, yielded a yellowish cheesy material along with hair shafts. The microscopic evaluation demonstrated cystic wall lined by mature squamous epithelium along with adjacent skin adnexal tissue, cartilage, fat, respiratory epithelium, and intestinal-type glandular tissue. Within intracystic spaces, a normal brain parenchymal tissue was identified; in addition, two foci of diverse glial neoplasms were noted (Figure 1(a)). The first neoplasm was composed of bipolar cells with elongated hair-like processes arranged in parallel bundles with prominent Rosenthal fibers and eosinophilic protein droplets. Histological features were classical for pilocytic astrocytoma grade 1 (Figure 1(b)). The second focus demonstrated a glial neoplasm comprised of clusters of isomorphic nuclei embedded in a subtle, dense, glial fibrillary background (Figure 1(c)). Occasional ependymal pseudorosettes were identified. No evidence of mitoses, necrosis, or endothelial proliferation was seen. Morphological features were more in keeping with subependymoma (Figure 1(d)). Ki67 proliferation index in both lesions was low (less than 1%). Immunohistochemistry for glial fibrillary acidic protein (GFAP) revealed diffuse and robust staining in both lesions (Figures 1(e) and 1(f)). S100 was strongly positive in the focus of subependymoma (Figure 1(g)). The final diagnosis was mature cystic teratoma with combined glial neoplasms (pilocytic astrocytoma grade 1 and subependymoma). Three months postoperatively, the patient was doing well, healthy, and alive.

## 3. Discussion

Glial and neuronal neoplasms arising in mature cystic teratoma of the ovary represent neuroectodermal-type tumors with similar morphology to their counterparts in the central nervous system (CNS). Neuroectodermal ovarian neoplasms are classified into a well-differentiated neuroepithelial component, primitive (immature) neuroepithelial tumors, and anaplastic tumors [11, 12]. The last two categories have unfavorable prognosis. Therefore, it is critical to identify immature primitive neuroepithelial elements in immature teratoma, which is currently preferably called “malignant ovarian teratoma” usually observed in children and adolescents [13, 14]. Pure malignant neuroectodermal tumor of the ovary is considered as a malignant form of monodermal teratoma. A distinct histopathological theme is an ovarian ependymoma, which is made up of primitive ependymal elements [15]. Solid mature teratomas are rare, occur primarily in children and young adults, and are considered WHO grade 0. In contrast, solid immature teratomas are represented by a mixture of immature tissue derived from the three germ layers. A careful gross examination is considered very important when handling specimens of ovarian teratomas with solid areas. Extensive tissue sampling of any solid areas would likely divulge morphologic features of a significant diagnostic and prognostic value, especially immature elements [16]. Therefore, we cannot emphasize enough the importance of good tissue sampling to exclude grade 1 immature teratoma. These two tumors are closely related; some authors believe that mature solid teratoma is “grade 0” immature teratoma [17]. Mature cystic teratomas may show cystic spaces and cavities lined by choroid plexus epithelium, neuronal ganglia, nerve bundles, and Schwann cell differentiation. Medulloblastoma, neuroblastoma, and gliomas are often classified as neuronal monodermal teratomas. It is uncommon to see glial neoplasm arising in ovarian teratoma. An extensive literature review reveals only 43 reported cases of different histologic subtypes of gliomas arising in both mature and immature ovarian teratoma. Some of these cases are reported as monodermal ependymal teratomas. Astrocytic neoplasms are considered the most commonly reported histologic variant among all gliomas. One study reported a series of malignant ovarian tumors, in which 13 cases were diagnosed as high-grade gliomas; one of them presented initially with metastatic tumors [18]. The remaining gliomas were reported as low-grade fibrillary astrocytoma (4 cases), pilocytic astrocytoma (1 case), and oligodendrogliomas (9 cases), and 16 cases were ependymomas (one of which was of myxopapillary ependymal neoplasm). Among these cases, seven were reported in immature teratomas (5 oligodendrogliomas and 2 glioblastomas). The remaining cases occurred in mature cystic teratoma. Furthermore, all ovarian ependymomas (except myxopapillary type) were reported as monodermal ependymal teratomas. None of the reported cases described a simultaneous occurrence of two different glial neoplasms in neither mature nor immature ovarian teratoma. The current case represents a unique pathological finding in which an area of classic pilocytic astrocytoma identified juxtaposed to a focus of subependymoma; both arise within mature cystic teratoma. The differential diagnosis includes epidermoid cyst, immature teratoma, gliomatosis peritonei, and peritoneal melanosis. Histologically, epidermoid cyst characteristically would lack skin adnexa. Immature teratoma typically demonstrates primitive neuroepithelial elements. Gliomatosis peritonei is a benign process composed of miliary-like grayish nodules on the peritoneal surface and omentum. It can be accompanied by chronic inflammation and fibrosis. Gliomatosis peritonei is composed of mature glial and neuronal tissue without any other teratomatous component. Peritoneal melanosis is another teratoma-related pathology, which can follow the rupture of a cystic teratoma component.

In summary, we report a unique case of collision glial tumors to arise in background mature cystic teratoma. To the best of our knowledge, this is the first reported case of concurrent pilocytic astrocytoma and subependymoma arising in an ovarian mature cystic teratoma occurring in a young female. Adequate tissue sampling is necessary to establish a precise histological diagnosis and appropriate evaluation.
